# Work Ability in the Digital Age: The Role of Work Engagement, Job Resources and Traditional and Emerging Job Demands Among Older White-Collar Workers

**DOI:** 10.3390/bs16020191

**Published:** 2026-01-29

**Authors:** Cristina Di Tecco, Ivan Marzocchi, Simone Russo, Anna Comotti, Alice Fattori, Marco Laurino, Pasquale Bufano, Catalina Ciocan, Luca Ferrari, Matteo Bonzini

**Affiliations:** 1 Department of Occupational and Environmental Medicine, Epidemiology and Hygiene, Italian Workers’ Compensation Authority (INAIL), 00078 Rome, Italy; 2 Department of Psychology, Sapienza University of Rome, 00185 Rome, Italy; 3 Occupational Medicine Unit, Foundation IRCCS Ca’ Granda Ospedale Maggiore Policlinico, 20122 Milan, Italy; 4 Department of Clinical Sciences and Community Health, University of Milan, 20122 Milan, Italy; 5 Institute of Clinical Physiology, National Research Council (CNR), 56124 Pisa, Italy; 6 Department of Public Health and Pediatrics, University of Turin, 10124 Turin, Italy

**Keywords:** aging, motivation, work characteristics, technostress creators, work ability

## Abstract

Aging may lead to a gradual decline in work ability, but other factors, such as worker motivation, also play a crucial role. This study, based on the Job Demands–Resources model, examined whether work engagement, an indicator of high motivation, is positively linked to work ability in older white-collar workers. We also explored the influence of job resources (control and social support) and demands (workload and techno-complexity) on work ability, mediated by work engagement. Structured interviews were conducted with 230 bank and finance workers aged over 50, and structural equation modeling was employed to investigate our hypotheses. Work engagement was positively associated with work ability. Control and social support improved work engagement and indirectly enhanced work ability. Among job demands, techno-complexity reduced work engagement but did not affect work ability. Workload weakened the positive effects of control on work engagement and work ability. Our findings suggest that promoting work engagement in older white-collar workers by increasing autonomy, fostering a supportive environment, and reducing overload and techno-complexity can help protect and enhance their work ability.

## 1. Introduction

The modern work landscape is undergoing a profound transformation driven by converging factors that shape individuals’ workforce experiences. These changes have resulted in the current world of work being characterized by significant shifts such as an aging workforce, technological advancements, and new ways of working. Understanding the intricate connections between these factors is essential for organizations aiming to create a supportive work environment (Schulte et al., 2019). With declining birth rates and increasing life expectancy in Western countries, the dynamics of a multigenerational workforce are evolving, extending careers and expertise requirements beyond traditional limits (Wang & Shi, 2014; World Economic Forum, 2023).

Aging can offer both opportunities and challenges. On the one side, older workers can bring a wealth of experience and knowledge to the table, which can be valuable for organizations (Warr, 1993). On the other side, aging is potentially linked to a gradual decline in work ability (Ilmarinen, 2012). As a result, maintaining work ability is crucial for ensuring sustained performance, well-being, and productivity throughout an individual’s career (Ilmarinen & Ilmarinen, 2015). Notably, variability in work ability exists due to factors beyond age (Converso et al., 2015). Therefore, further research is necessary to identify the individual and work attributes that influence work ability among older workers, especially in a transforming workplace shaped by the integration of Information and Communication Technologies (ICTs). While these technologies aim to enhance work experiences, they have also led to unintended challenges for older employees (Nimrod, 2018). Such challenges may be especially pronounced in certain sectors, such as banking and financial services, which have become highly digitalized environments through the widespread adoption of ICTs to streamline operations, improve customer service, and ensure regulatory compliance (Porfírio et al., 2024).

**Figure 1:**
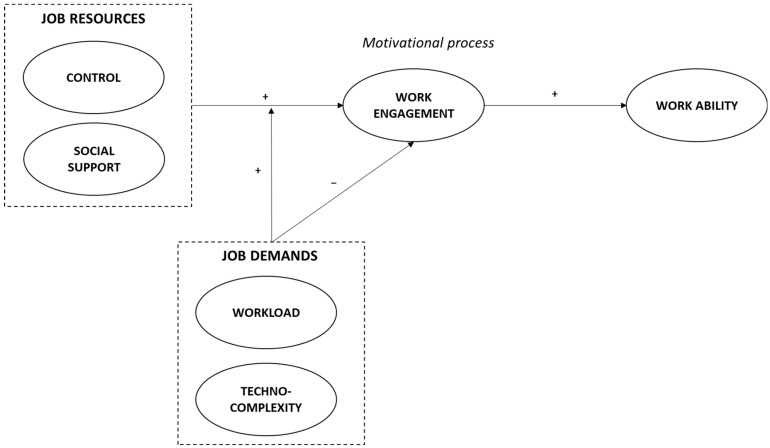
The posited model.

Drawing on the Job Demands–Resources (JD-R) model as a theoretical framework (Bakker & Demerouti, 2017), the current study explores the role of workers’ motivation and work characteristics in affecting work ability among older workers. We aim (a) to explore the role of work engagement in influencing work ability among older white-collar workers, and (b) to investigate whether traditional and emerging work characteristics may affect their work ability by increasing or reducing work engagement. Through this study, we extend the existing literature on work ability among older workers by investigating whether work engagement, which refers to a positive behavior or a positive mental state at work (Bakker & Demerouti, 2017), may be an antecedent of work ability. Past studies have shown that work engagement is an essential attribute for employee well-being and performance (Mazzetti et al., 2023). Despite this, only a limited number of studies have examined whether work engagement, as a critical indicator of high worker motivation, may also contribute to maintaining work ability among older workers. Additionally, we offer new insights into both the independent and interactive effects of work characteristics on employee motivation and, consequently, work ability, by examining their unique contributions as well as their interplay through moderation mechanisms. Notably, in today’s fast-paced work environment, it is crucial to have up-to-date understanding of how both traditional and emerging challenges negatively affect work ability, especially when work engagement is low. Equally important is identifying the job resources that can boost older workers’ motivation, thereby maintaining or enhancing work ability. These insights can help organizations support work ability throughout a worker’s entire career. Figure 1 illustrates our posited model.

### 1.1. Work Ability, Work Engagement and Work Characteristics

Aging is a continuous process and there is no universal agreement on the specific age at which workers are considered “older” (Zacher et al., 2018). In this study, the threshold of 50 years is used to define older workers, a common benchmark in the literature (Jones et al., 2013; de Lange et al., 2006). Additionally, we focus on white-collar jobs and the banking and finance sector as they are more often associated with digitalization and new ways of working (Dingel & Neiman, 2020; Porfírio et al., 2024). What is generally recognized is that aging is potentially linked to declines in both physical and cognitive functioning (Peng & Chan, 2019). Nevertheless, the extent of this decline varies significantly based on individual factors such as lifestyle and physical activity. These differences can influence the balance between a worker’s capacity and job requests: when this balance is misaligned, particularly because of aging, health issues may arise, which can ultimately push workers out of the workforce (Woźniak et al., 2022). Given that work-related health problems are a major cause of unemployment and early retirement among older workers, it is crucial to focus on maintaining and improving work ability. This includes not only understanding the causes of reduced work ability, but also identifying the factors that can protect or enhance it.

Work ability can be described as an individual’s self-assessment of their capacity to meet the demands of their job (McGonagle et al., 2015). Poor work ability predicts withdrawal behaviors such as sickness absences, early retirement, and turnover intention (Kinnunen & Nätti, 2018). Conversely, good work ability is linked to positive outcomes such as productivity and well-being (Camerino et al., 2006). Poor work ability resulting from age-related decline may be attributed to a general increase in diseases over the lifespan: as individuals age, they may become more susceptible to both physiological and psychological health conditions (Blane et al., 2008). However, although work ability is first a question of balance between work demands (physical and/or psychological) and personal characteristics (Ilmarinen et al., 2005), other key factors have been identified as pivotal in determining the extent of work ability, including the workers’ motivation (Ilmarinen, 2019).

Among the indicators of workers’ motivation, work engagement refers to a positive mental state associated with work, marked by a high level of energy and resilience (vigor), a strong sense of connection to one’s work (dedication), and a favorable state of deep immersion and focus on work tasks (absorption) (Bakker & Demerouti, 2017). Previous studies underscored its prominent role in enhancing job performance and enriching well-being (Mazzetti et al., 2023). Since work engagement holds significant importance in today’s dynamic and evolving work environments, identifying which work characteristics may activate it becomes essential for promoting both individual well-being and organizational success. In line with the JD-R model (Bakker & Demerouti, 2017), work characteristics are considered as potential antecedents of work engagement. These can be grouped into two primary categories: job resources and job demands.

Job resources are those physical, psychological, social, or organizational aspects of the work context that can reduce job demands and their straining impact, are functional in achieving work goals, stimulate personal growth and, thus, determine well-being (Bakker & Demerouti, 2017). Among job resources, control and social support are considered as some of the most effective work features to improve worker’s well-being and performance (Van der Doef & Maes, 1999). Control refers to the degree to which workers have autonomy over their work and can make decisions independently. Concurrently, social support represents the degree to which workers feel supported by their colleagues and managers (Karasek & Theorell, 1990).

Conversely, job demands are those physical, psychological, social, or organizational aspects of the work context associated with certain psychological and/or physiological costs (Bakker & Demerouti, 2017). In the current study we focus on both a traditional job demand (i.e., workload) and an emerging one (i.e., techno-complexity). Workload, a demanding aspect of the job that includes the pressure for immediate decisions, increased responsibilities, and tight deadlines, is known to decrease workers’ well-being (Bowling et al., 2015). To follow, techno-complexity is an important source of distress among older employees (Nimrod, 2018). Techno-complexity refers to the feeling of inadequacy when older employees encounter challenges related to ICTs. As a result, they feel forced to invest substantial time and effort into learning and navigating the various functions of these technologies (Nimrod, 2018).

### 1.2. Association Between Job Resources, Work Engagement and Work Ability

Job resources yield favorable outcomes for employee well-being and performance through work engagement, a mechanism activated by the so-called motivational process (Bakker & Demerouti, 2017). Control and social support are among the most important antecedents of work engagement (Mazzetti et al., 2023; Van der Doef & Maes, 1999). Their role appears to be relevant across the working lifespan; however, some evidence suggests that their influence may be less pronounced among older and more experienced workers. On the one hand, older workers might highly value autonomy and positive social interactions, appreciating an organization that offers freedom and engagement opportunities. On the other hand, compared to younger workers, they may derive fewer additional benefits from high levels of control or support, given their greater experience, established work strategies, and higher self-regulatory capacities (Ng & Feldman, 2015; Stawski et al., 2008). Thus, while control and social support remain important resources regardless of age, their incremental contribution to work engagement may be less evident among more experienced workers.

Additionally, while the association between work engagement and workers’ health and performance is better understood (Mazzetti et al., 2023), its relationship with work ability has received scarce attention and little exploration. Some findings have revealed that those exhibiting higher levels of work engagement may also demonstrate elevated work ability over time. For example, Airila et al. (2012) found that work engagement was longitudinally associated with increased work ability in a sample of firefighters. Rongen et al. (2014) found that low work engagement was related to reduced work ability, beyond the effects of health behaviors and work-related characteristics. Airila et al. (2014) showed that work engagement fully mediated the influence of job and personal resources on work ability, highlighting the work engagement’s contribution to enhancing work ability over time. Similarly, Debets et al. (2022) found that work engagement mediated the relationship between several job resources (i.e., development opportunities, decision-making, and workplace relationships) and work ability, so that more engaged workers reported better work ability.

Notably, to our knowledge, only one study has been conducted focusing on older workers, with non-conclusive results (Tomietto et al., 2019). The authors found that certain aspects of work engagement, such as dedication, may be related to work ability in older workers, whereas others, like vigor, may be more advantageous for younger workers. They also suggested that the absorption dimension of work engagement, which reflects deep involvement and focus on work, could be seen negatively by older workers in relation to work ability. This negative view may stem from the risk of burnout, particularly when work–life balance is not maintained.

Taken together, these findings suggest that the relationship between job resources, work engagement, and work ability may be more nuanced in later career stages. These results do not call into question the central role of job resources and work engagement as key motivational ingredients. Rather, they point to the need for a deeper understanding of the mechanisms through which job resources enhance work engagement and, in turn, support work ability among older workers. Within this framework, and in line with the assumptions of the JD-R model, the following hypotheses are proposed:

**H1.**  *Control and social support are positively associated with work engagement;*

**H2.**  *Work engagement is positively associated with work ability;*

**H3.**  *Work engagement mediates the association between control and social support and work ability.*

### 1.3. Association Between Job Demands, Work Engagement and Work Ability

Previous research has highlighted that job demands can diminish work ability across various work environments (Airila et al., 2014; Bernburg et al., 2016), especially through the onset of burnout (Debets et al., 2022). Stressors such as workload and techno-complexity are believed to initiate a process of energy depletion (Demerouti et al., 2001), thus leading to the exhaustion of essential resources and the drainage of workers’ energy. In particular, the perceived complexity of using technology (i.e., techno-complexity) is often perceived as a barrier for older workers: when technology is too complex, it can make older workers feel incompetent, leading them to spend more time and effort trying to understand it (Nimrod, 2018). This can create a negative feedback loop, as the more time older workers spend trying to understand the technology, the more inadequate, stressed, and demotivated they may feel (Nimrod, 2018). Consequently, workload and techno-complexity may increase strain, undermining workers’ motivation and their ability to effectively fulfill job duties.

Notably, there has been limited empirical investigation into the role of job demands as antecedents of work engagement and work ability, with often mixed results. First, research has prioritized the examination of job and personal resources over job demands (Airila et al., 2014). Second, as in Debets et al. (2022), job demands have predominantly been studied as initiators of the health impairment process, which links these potentially harmful factors to burnout (Demerouti et al., 2001). Third, job demands have been shown to be more strongly associated with strain-related outcomes than with motivational outcomes. For instance, a meta-analysis by Bowling et al. (2015) demonstrated that workload was positively related to negative indicators such as strain, depression, and burnout, while showing no significant association with work engagement. This result was further corroborated by Mauno et al. (2013), who reported no significant association between workload and work engagement, regardless of participants’ age. Finally, Crawford et al. (2010) found that although all job demands are potentially stressful and energy-depleting, only specific types of demands (i.e., hindrance job demands) were negatively associated with work engagement, whereas others (i.e., challenge job demands) were positively or not associated with it.

Nevertheless, from a lifespan and aging-at-work perspective, both excessive workload and techno-complexity may represent significant work-related obstacles for older workers (Nimrod, 2018; van der Meer et al., 2016). Age-related changes in cognitive resources and adaptability to technological change may intensify the detrimental impact of these job demands, thereby undermining motivational processes. Accordingly, we formulated the following hypotheses:

**H4.**  *Workload and techno-complexity are negatively associated with work engagement;*

**H5.**  *Work engagement mediates the association between workload and techno-complexity and work ability.*

On a final note, job resources and job demands may exert joint effects on employee outcomes; however, empirical evidence regarding their interaction has often been mixed and sometimes contradictory (Parker et al., 2017). According to the “boosting hypothesis”, postulated by the JD-R model, moderate to high levels of job demands may strengthen the motivational potential of job resources, thereby amplifying their positive effects on work engagement and related outcomes (Marzocchi et al., 2023). This occurs because abundant job resources may help workers view challenges like work overload and new technologies as growth opportunities (Bakker & Demerouti, 2017). However, some research suggests that optimal motivation may not necessarily emerge in contexts characterized by simultaneously high job demands and abundant resources. Instead, more favorable motivational outcomes have been observed in comparatively less demanding work environments (Holman & Wall, 2002). Moreover, other studies have failed to find a significant moderating role of job demands in the relationship between job resources and motivational outcomes, further underscoring the inconsistency of empirical evidence in this area (Ouweneel et al., 2009).

Despite these mixed findings, we aim to test the boosting hypothesis by examining whether job demands strengthen the positive effects of job resources on work engagement and work ability among older workers. Specifically, we propose that high levels of workload and techno-complexity may amplify the beneficial impact of job control and social support. Accordingly, the following hypotheses are formulated:

**H6.**  *Job demands moderate the relationship between job resources and work engagement, such that higher levels of workload and techno-complexity strengthen these associations;*

**H7.**  *Job demands moderate the indirect relationship between job resources and work ability through work engagement, such that higher levels of workload and techno-complexity strengthen these indirect associations.*

## 2. Materials and Methods

### 2.1. Participants and Procedure

Data were obtained from a cross-sectional observational study conducted among white-collar workers aged over 50 employed in the banking and finance sector. The comprehensive study protocol has been previously published (Bonzini et al., 2023). During the medical monitoring required by the Italian Legislative Decree 81/08 and its subsequent amendments, the occupational physician invited workers to participate in the study. Those who furnished written consent were subsequently included and designated with pseudo-anonymous identifiers. Participants’ information was collected using REDCap, a web-based platform developed by Vanderbilt University for data collection in clinical research and for establishing databases and projects. Data collection was performed between November 2021 and November 2022. This study specifically focused on 230 workers (63.5% males, 36.5% females, 80% of response rate)^1^. Most of the participants were aged between 50 and 55 (49.3%), 41% were aged between 56 and 60, and 9.6% were aged more than 60 (mean age = 55.51; SD = 3.56). The majority had a job tenure from 31 and 35 years (43%) and up to 30 years (32.6%), while 24.3% had a job tenure of more than 35 years (mean job tenure = 32.24; SD = 4.62). Most of participants worked remotely and in presence (94.8%), with a mean percentage of weekly remote work of 52% (SD = 17.92); 5.2% of participants worked only in presence.

We performed a check of missing data on all the variables considered in this study. Little’s test of missing data was not significant (χ^2^(126) = 102.33, *p* = 0.94), suggesting that data were missing completely at random (MCAR).

### 2.2. Measures

Workload was measured using three items from the subscale “Demands” of the Italian version of the Management Standards Indicator Tool (MS-IT) (Rondinone et al., 2012). A sample item was “I have unachievable deadlines”.

Techno-complexity was assessed with three items from a technostress scale developed exclusively for use among older adults (Nimrod, 2018), validated in Italian by Comotti et al. (2025). An item example was “I often find the technology too complex to use”.

Control was measured through three items from the MS-IT (Rondinone et al., 2012) reflecting the autonomy that workers had in exercising their own work activities. An item example was “I have a choice in deciding how I do my work”.

Social support was assessed using six items from the MS-IT (Rondinone et al., 2012) describing the encouragement and support provided by colleagues and/or supervisors. A sample item was “I get help and support I need from colleagues”.

Work engagement was measured through the ultra-short version of the Utrecht Work Engagement Scale (UWES-3) (Schaufeli et al., 2019). A sample item was “At my work, I feel bursting with energy”.

Work ability was assessed through the Work Ability Index (WAI) (Tuomi et al., 2006). The WAI is a tool used in occupational health to monitor and assess how well employees are able to carry out their job tasks during routine medical examinations and workplace assessments. To do this, the WAI explores seven main dimensions: (1) the worker’s current ability to work compared with their best level ever; (2) work ability in relation to job demands; (3) the number of medically diagnosed conditions; (4) the perceived impact of these conditions on work performance; (5) the amount of sick leave taken in the previous year; (6) the individual’s own forecast of their work ability two years before the assessment; and (7) an evaluation of mental resources.

Employees were asked to answer questions on job demands (workload, techno-complexity) and resources (control, social support) using a five-point Likert scale (1 = strongly disagree to 5 = strongly agree), while for work engagement they used a seven-point Likert scale (1 = never to 7 = always). Regarding work ability, the overall score for the seven dimensions ranges from 7 to 49, with higher values reflecting greater work ability.

### 2.3. Analytic Strategy

We first examined the factor structure of the variables using Confirmatory Factor Analysis (CFA) using a full information maximum likelihood (FIML) estimation strategy (Arbuckle, 1996). Workload, techno-complexity, control, and work engagement were treated as latent variables measured by their respective items, while social support was defined as a latent variable measured by two parcels: management support and colleagues’ support. Conversely, the total WAI score was treated as an observed variable and calculated according to the standard procedure provided by the Finnish Institute of Occupational Health (Tuomi et al., 2006). We included gender (1 = males, 2 = females), percentage of remote working per week, and job tenure as control variables in all the analyses. To assess model fit, we used the Yuan–Bentler (YB) Chi-square test, Comparative Fit Index (CFI), Tucker and Lewis Index (TLI), Root Mean Square Error of Approximation (RMSEA), and Standardized Root Mean Square Residual (SRMR). The following cutoff values were adopted: CFI ≥ 0.90, TLI ≥ 0.90, RMSEA ≤ 0.08, and SRMR ≤ 0.08 (Hu & Bentler, 1999).

After confirming the measurement model’s fit, we tested our moderated–mediated model using a latent moderation structural equation model (LMS) following a multi-step approach (Sardeshmukh & Vandenberg, 2017). First, we tested a baseline model without latent interaction terms (Model 1), which included the direct effects of workload, techno-complexity, control, and social support on work engagement, as well as the effect of work engagement on work ability^2^. The mediation hypotheses were tested using the “Model Indirect” procedure in Mplus, with 95% Bootstrapped Confidence Intervals (CIs) calculated using 5000 samples (MacKinnon et al., 2007).

We then evaluated four LMS models, each testing a specific latent interaction term. A final model, including only significant interactions, was estimated, and conditional indirect effects were computed. As common fit indices are not available for LMS models, we assessed the overall fit by employing the log-likelihood ratio test (−2[(log-likelihood for the baseline model) − (log-likelihood for the LMS model)]) (Sardeshmukh & Vandenberg, 2017). This test allowed us to determine whether the more parsimonious model without interaction terms exhibited a significant loss of fit compared to the LMS model (Maslowsky et al., 2015). Additionally, to corroborate the log-likelihood ratio test results, we compared AIC values between the baseline and LMS models (ΔAIC = AIC for LMS model − AIC for baseline model), preferring models with the lowest AIC values (Vandenberg & Grelle, 2009).

All analyses were conducted using IBM SPSS v.23 and Mplus 8.7 (Muthén & Muthén, 2017).

## 3. Results

### 3.1. Descriptive Statistics and Measurement Model

Table 1 presents descriptive statistics and correlations among study variables. Cronbach’s alphas were all adequate (>0.70). Workload correlated positively with techno-complexity and negatively with control, social support, and work engagement. Techno-complexity correlated negatively with social support, work engagement, and work ability. Control, social support, work engagement, and work ability were positively correlated with each other.

Control, social support, and work ability had skewness/kurtosis values outside −1 to +1, so the Robust Maximum Likelihood (MLR) estimator was used to address non-normality (Yuan et al., 2000). The measurement model fit well: YBχ^2^(67) = 106.353, *p* < 0.001; CFI = 0.950; TLI = 0.932; RMSEA = 0.051 (95%CI: 0.031, 0.068; *p* = 0.461); SRMR = 0.046. Factor loadings ranged from 0.50 to 0.86. ($\bar{\lambda }$ = 0.73; SD = 0.11).

### 3.2. Mediation Model

The mediation model, in which job demands and resources influenced work engagement, which in turn affected work ability, demonstrated good fit: YBχ^2^(107) = 171.652, *p* < 0.001; CFI = 0.931; TLI = 0.903; RMSEA = 0.051 (90%CI: 0.037, 0.065; *p* = 0.427); SRMR = 0.050. In line with H1, control (β = 0.43, *p* < 0.001) and social support (β = 0.23, *p* < 0.05) were significantly associated with work engagement. Consistent with H2, work engagement was positively related to work ability (β = 0.30, *p* < 0.001). Supporting H3, control (β = 0.129, Bootstrap 95% CI: 0.043, 0.232) and social support (β = 0.070, Bootstrap 95% CI: 0.004, 0.144) were indirectly associated with work ability via work engagement.

H4 received partial support: techno-complexity was negatively associated with work engagement (β = −0.17, *p* < 0.05), whereas workload was not (β = −0.02, *p* = 0.88). H5 was not supported, as techno-complexity was not significantly linked to work ability through engagement (H3b; β = −0.051, Bootstrap 95% CI: −0.118, 0.002), and the mediation path from workload to work ability through work engagement was not tested due to the non-significant association between workload and work engagement.

### 3.3. Moderated–Mediated Model

**Figure 2:**
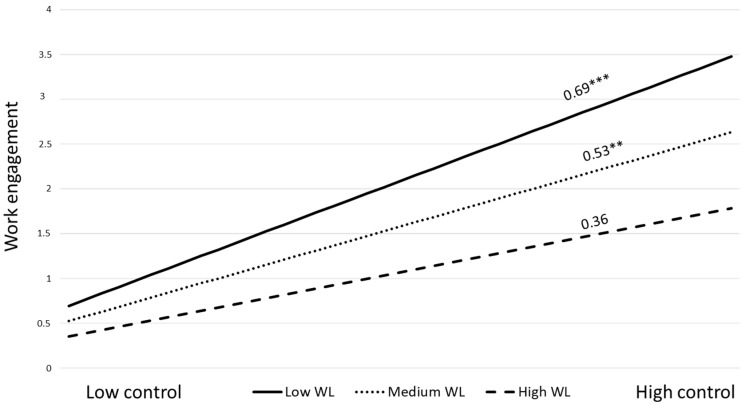
Moderating effect of workload on the control–work engagement path (N = 230; WL = workload. *** *p* < 0.001, ** *p* < 0.01).

Table 2 presents the results of the four models with latent interactions. The results show a significant moderating effect of workload on the relationship between control and work engagement (Model 1a). However, contrary to our expectations, workload buffered the association between control and work engagement (β = −0.14, *p* < 0.05). The simple slope analysis (Figure 2) indicated that the relationship between control and work engagement was stronger for workers with low (b = 0.69, *p* < 0.001) and medium levels of workload (b = 0.53, *p* < 0.001), while was not significant for those with high workload (b = 0.36, *p* = 0.053). Thus, H6 was not supported.

**Figure 3:**
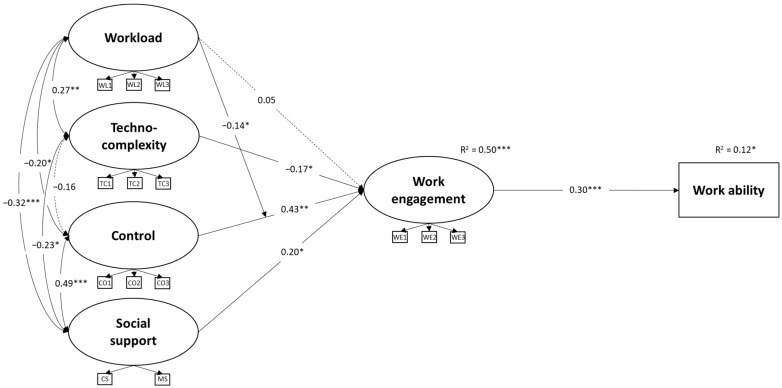
Results of the final model with significant latent interactions (N = 230; dotted lines denote not-significant paths; percentage of remote working, gender and job tenure have been included as control variables). *** *p* < 0.001, ** *p* < 0.01, * *p* < 0.05.

Figure 3 shows the results of the final model (Model 1a) including the significant latent interaction. All the other relationships estimated in the model were consistent with those observed in the mediation model described above. The additional variance explained in work engagement by the interaction terms was 5.2%. H7 was not supported, as workload attenuated rather than enhanced the indirect effect of control on work ability through work engagement. Indeed, control was associated with work ability through work engagement at low (b = 0.724, *p* < 0.01) and medium workload levels (b = 0.548, *p* < 0.05) (Table 3), but not at high workload levels (b = 0.371, *p* = 0.109).

Among the covariates, remote working was negatively related to workload (β = −0.20, *p* < 0.05) and positively related to control (β = 0.18, *p* < 0.05) and work engagement (β = 0.13, *p* < 0.05), indicating that individuals who worked more frequently remotely reported lower workload, higher control, and greater work engagement. Female gender was associated with higher workload (β = 0.19, *p* < 0.01), while longer job tenure was associated with reduced work ability (β = −0.16, *p* < 0.01).

## 4. Discussion

### 4.1. Theoretical Implications

While studies on work engagement have increased (Mazzetti et al., 2023), research focusing on its association with work ability, especially among older workers, remains scarce. The present findings support the role of work engagement as a relevant predictor of work ability among older white-collar workers, and indicate that job resources, rather than job demands, act as the primary drivers of this process.

In line with the JD-R model main underpinnings, control and social support were positively linked to work engagement (Bakker & Demerouti, 2017). In turn, they exerted an indirect positive influence on work ability (Airila et al., 2014). Thus, job resources such as control and social support may have a significant impact not only on positive outcomes, such as improved job performance and health (Bakker & Demerouti, 2017), but also on work ability. Consequently, work environments characterized by supportive conditions, including individual discretion in skills and decision-making, positive interactions among colleagues, and optimal managerial support, have the potential to cultivate thriving and engaged workers who enjoy strong work ability (Airila et al., 2014). Our results are consistent with the Self-Determination Theory (SDT), which states that all humans have specific basic psychological needs which want to satisfy, including autonomy (i.e., control) and relatedness (i.e., social support) (Ryan & Deci, 2000). When these needs are met, people are more likely to be motivated in a self-determined way, something that is associated with positive outcomes such as well-being and productivity. Transferring these insights to our study, when older workers have autonomy and feel supported at work, they are more likely to be motivated to do their best at work; this has positive implications for their work ability.

Our results underlined that job demands may have detrimental effects on work engagement. In particular, we found that techno-complexity (namely, the feeling that the technological environment is very complex) is an emerging risk factor that could have a direct negative influence on work engagement among older white-collar workers. These detrimental effects may be especially pronounced in the banking and finance sector, where employees are required to interact with a wide array of information systems, including core banking platforms, customer relationship management systems, compliance and regulatory software, and digital communication tools (Porfírio et al., 2024). This constant flow of information and the need to manage multiple tasks simultaneously may result in increased fatigue, diminished concentration, and a higher risk of burnout (Karr-Wisniewski & Lu, 2010). In this context, our results suggest that older workers may experience greater difficulties in processing new and complex information, which may in turn undermine their motivation (Nimrod, 2018). However, techno-complexity did not exert an indirect negative influence on work ability. As described in the introduction, and since job demands are the main initiators of the health impairment process (Bakker & Demerouti, 2017), we may suggest that these stressful conditions could reduce workers’ work ability especially through increased strain (e.g., burnout, negative emotions) rather than reduced well-being (e.g., work engagement).

In a similar vein, and contrary to our expectations, we found workload to be not significantly associated with work engagement. Prior studies reported contradictory results on that path, with some of these suggesting positive, negative, and non-significant relationships among the two constructs (Bowling et al., 2015; Hakanen et al., 2008). One possible reason is that workload may have specific effects depending on how it is perceived by workers (“challenges” or “hindrances”) (LePine, 2022). Thus, future studies should explore deeply how older workers appraise specific stressors, such as workload.

Finally, our results provided limited support for the interactions between job demands and resources, as all these effects (except one) were not significant. Additionally, contrary to the boosting hypothesis, the only significant interaction suggested a buffering rather than an enhancing role of workload in the association between control and work engagement. In particular, at high levels of workload, the indirect effect of control on work ability through work engagement was no longer statistically significant. These results may indicate that, even when older workers have access to substantial job resources such as control and social support, high workload is not necessarily experienced as a motivating challenge. Instead, excessive workload might reduce the extent to which job resources translate into higher work engagement and, consequently, into better work ability. This pattern could be tentatively interpreted in light of age-related changes in priorities and personal resources, such as lower energy availability, a stronger focus on maintaining work–life balance, and a more limited time horizon to benefit from learning and growth opportunities as retirement approaches (Peng & Chan, 2019; Richert-Kaźmierska & Stankiewicz, 2016).

### 4.2. Practical Implications

Older workers contribute valuable experience and knowledge to organizations; thus, it is crucial to develop strategies for sustaining workforce engagement and work ability throughout their entire working lives (World Health Organization, 2002). Our research highlights the importance of work engagement in fully mediating the relationship between job resources and work ability among older white-collar workers. Thus, organizational interventions promoting work engagement, such as providing autonomy, fostering collaboration, and creating supportive work environments, could be particularly effective to protect and promote older workers’ work ability (Knight et al., 2017). Additionally, our results underline that organizations should properly address techno-complexity and its impact on older workers. Digitalization has introduced new risk factors, including not only techno-complexity but also other techno-stressors (e.g., techno-overload), blurred boundaries between work and private life due to constant connectivity, and increased social isolation (e.g., Nastjuk et al., 2024). Thus, understanding their specific impact on older workers is crucial for developing mitigation strategies. Providing additional training, user-friendly technologies, and accessible designs are potential solutions to overcome the contingent detrimental effects of technology for this category of workers. In a similar way, interventions such as setting realistic work schedules, ensuring physical and psychological recovery, and decreasing long working hours may be useful to mitigate the negative effects of excessive workload.

### 4.3. Limitations and Future Research Directions

Our study has several limitations that should be acknowledged. First, the cross-sectional design limits causal inferences. While work engagement is often viewed as a precursor to work ability, this relationship may be bidirectional, with work ability also influencing engagement (Cadiz et al., 2019). There is similarly a reciprocal relationship between work engagement, job demands, and resources (Lesener et al., 2019). Future research should utilize longitudinal designs to explore these relationships over time.

Second, our sample’s specific characteristics limit the generalizability of the findings. Conducted in Italy, one of Europe’s fastest-aging countries (Istituto Nazionale di Statistica, 2022), the study used a convenience sample of bank and finance workers, lacking a comparison group of younger or middle-aged workers. This raises questions about the age-specific nature of the observed relationships. Future research should encompass diverse countries, work settings, and age groups for broader applicability.

Third, reliance on self-reported measures may introduce common method and social desirability biases (Podsakoff et al., 2003). Future studies should include objective measures from various sources, such as actual working hours and organizational data, to mitigate this limitation.

## 5. Conclusions

This study explores the interplay between work characteristics, work engagement, and work ability among older white-collar workers. Our findings confirm the pivotal role of work engagement in positively influencing work ability, demonstrating how dedication, task absorption and vigor contribute to sustaining older workers’ capacity to perform effectively. Consistent with the JD-R model, job resources such as control and social support emerge as significant facilitators of work engagement, indirectly enhancing work ability. Conversely, our results underscore the challenges posed by job demands, particularly techno-complexity, as an emerging risk factor negatively affecting work engagement or weakening the beneficial effects of job resources. These findings underscore the importance of tailored interventions to address the unique needs of older workers navigating increasingly digital work environments. Such interventions may include age-sensitive job design aimed at balancing job demands and resources, targeted training and support to reduce technostress and facilitate adaptation to digital tools, and initiatives that strengthen autonomy, social support, and opportunities for meaningful work engagement.

## Figures and Tables

**Table 1:** Descriptive statistics and correlations among study variables (N = 230).

Variable	Mean	SD	Sk	Ku	1.	2.	3.	4.	5.	6.
1. Workload	1.81	0.79	0.89	0.04	0.73					
2. Techno-complexity	2.38	0.64	0.84	0.64	0.21 **	0.73				
3. Control	3.69	0.66	−0.91	1.69	−0.15 *	−0.11	0.77			
4. Social support	4.04	0.41	−0.32	3.94	−0.26 ***	−0.17 **	0.36 ***	0.83		
5. Work engagement	4.21	1.00	−0.22	−0.04	−0.21 **	−0.20 **	0.47 ***	0.39 ***	0.85	
6. Work ability	44.74	2.90	−1.29	2.93	−0.13	−0.24 ***	0.26 ***	0.23 ***	0.25 ***	-

SD = standard deviation; Sk = skewness; Ku = kurtosis; Cronbach’s alphas are reported in diagonal. *** *p* < 0.001, ** *p* < 0.01, * *p* < 0.05.

**Table 2:** Results of the models tested (N = 230).

	#Parameters	Log-Likelihood	AIC	Log-Likelihood Test	ΔAIC	Interaction Term
Model 1	73	−3981.784	8109.568	-	-	-
Model 1a	74	−3978.762	8105.524	6.044 *	4.044	β = −0.14 *
Model 1b	74	−3980.651	8109.302	2.266	0.266	β = −0.11
Model 1c	74	−3981.466	8110.931	0.636	−1.363	β = −0.05
Model 1d	74	−3980.060	8108.121	3.448	1.447	β = −0.14

Model 1: baseline model with no interaction terms; Model 1a: interaction between control and workload on work engagement; Model 1b: interaction between control and techno-complexity on work engagement; Model 1c: interaction between social support and workload on work engagement; Model 1d: interaction between social support and techno-complexity on work engagement; * *p* < 0.05.

**Table 3:** Conditional indirect effects of control on work ability through work engagement (N = 230).

	Control → Work Engagement → Work Ability		
		Unstandardized Beta	*p*
Workload	Low (−1 SD)	0.724	<0.01
	Medium	0.548	<0.05
	High (+1 SD)	0.371	0.109

## Data Availability

The data that support the findings of this study are available from the corresponding authors upon reasonable request.

## Notes

1 Following the observations of an anonymous reviewer, we conducted a post hoc RMSEA-based power analysis to assess whether our sample size was adequate for the planned analyses using the web-based interface power4SEM (Jak et al., 2021). Specifically, with 107 degrees of freedom and a sample of 230 participants, power to reject the null hypothesis was 0.984 for RMSEA = 0.08 (hypothesis of close fit), 0.946 for RMSEA = 0.01 (hypothesis of not-close fit), and 0.963 for RMSEA = 0.00 (hypothesis of exact fit). These results indicate that the planned analyses could be reliably conducted.

2 Because our data were cross-sectional, we conducted an additional analysis by testing an alternative model in which workload, techno-complexity, control, and social support predicted work ability, which in turn predicted work engagement. However, this alternative model did not show an acceptable fit, as all goodness-of-fit indices indicated inadequate or poor model performance: YBχ^2^(107) = 225.765, *p* < 0.001; CFI = 0.873; TLI = 0.822; RMSEA = 0.069 (90% CI: 0.057–0.082; *p* < 0.01); SRMR = 0.095. These findings suggest that the model positioning work engagement as the mediator and work ability as the outcome provides a better fit for our data.

## Abbreviations

The following abbreviations are used in this manuscript:

ICTs	Information and Communication Technologies
JD-R	Job Demands–Resources
REDCap	Research Electronic Data Capture
MCAR	Missing Completely At Random
MS-IT	Management Standards Indicator Tool
UWES-3	Utrecht Work Engagement Scale-3 items
WAI	Work Ability Index
CFA	Confirmatory Factor Analysis
FIML	Full Information Maximum Likelihood
YB	Yuan–Bentler
CFI	Comparative Fit Index
TLI	Tucker–Lewis Index
RMSEA	Root Mean Square Error of Approximation
SRMR	Standardized Root Mean Square Residual
AIC	Akaike Information Criterion
LMS	Latent Moderation Structural Equation Model
SDT	Self-Determination Theory
MLR	Robust Maximum Likelihood
